# MicroRNAs as prognostic molecular signatures in renal cell carcinoma: a systematic review and meta-analysis

**DOI:** 10.18632/oncotarget.5324

**Published:** 2015-09-22

**Authors:** Liangyou Gu, Hongzhao Li, Luyao Chen, Xin Ma, Yu Gao, Xintao Li, Yu Zhang, Yang Fan, Xu Zhang

**Affiliations:** ^1^ Department of Urology/State Key Laboratory of Kidney Diseases, Chinese PLA General Hospital/PLA Medical School, Beijing 100853, P.R. China

**Keywords:** microRNA, renal cell carcinoma, survival, prognosis biomarker, meta-analysis

## Abstract

This is a systematic review of studies investigating the prognostic value of different microRNAs (miRs) in renal cell carcinoma (RCC). Twenty-seven relevant studies were identified, with a total of 2578 subjects. We found that elevated expression of miR-21, miR-1260b, miR-210, miR-100, miR-125b, miR-221, miR-630, and miR-497 was associated with a poor prognosis in RCC patients. Conversely, decreased expression of miR-106b, miR-99a, miR-1826, miR-215, miR-217, miR-187, miR-129–3p, miR-23b, miR-27b, and miR-126 was associated with a worse prognosis. We performed meta-analyses on studies to address the prognostic value of miR-21, miR-126, miR-210, and miR-221. This revealed that elevated miR-21 expression was associated with shorter overall survival (OS; hazard ratio [HR], 2.29; 95% confidence interval [CI], 1.28–4.08), cancer specific survival (CSS; HR, 4.16; 95% CI, 2.49–6.95), and disease free survival (DFS; HR, 2.15; 95% CI, 1.16–3.98). The decreased expression of miR-126 was associated with shorter CSS (HR, 0.35; 95% CI, 0.15–0.85), OS (HR, 0.45; 95% CI, 0.30–0.69), and DFS (HR 0.30; 95% CI, 0.18–0.50). Our comprehensive systematic review reveals that miRs, especially miR-21 and miR-126, could be promising prognostic markers and useful therapeutic targets in RCC.

## INTRODUCTION

A total of 63,920 new kidney and renal pelvis cancers were estimated to occur in the United States in 2014, and 13,860 deaths were related to these malignancies [1]. Epidemiologic data have shown a rapid rise in the incidence of renal cell carcinoma (RCC) [2]. RCC is the most common form of kidney cancer in adults, and is comprised of four major histologic subtypes, clear cell RCC (ccRCC), papillary RCC, chromophobe RCC, and oncocytomas. ccRCC remains the most aggressive and common subtype of RCC, accounting for 75% to 80% of cases [3]. Approximately 20–30% of RCC patients have metastatic disease at the time of diagnosis, and another 30% who undergo curative surgery for localized RCC develop metastasis during follow-up [4]. Hence, a means of identifying patients with a poor prognosis, and who may benefit from aggressive treatment, is greatly needed. The currently used system to predict prognosis is based on clinicopathological parameters [5], but does not accurately stratify patients, or predict the natural outcome of the disease, especially in localized RCC [6]. Therefore, molecular biomarkers that can improve the accuracy of predictions when used alone or in combination with other clinical parameters are urgently needed to better guide clinical decisions.

Although there has been widespread research into genetic biomarkers for RCC, epigenetic biomarkers including microRNAs (miRs) have also received considerable attention because of their biological and clinical utility in diagnosis and treatment [7]. MiRs are a class of small (∼22 nucleotide) noncoding RNAs that regulate post-transcriptional gene expression epigenetically, through RNA interference. This is usually mediated by their direct interaction with the 3′-UTR of complementary mRNA target transcripts, which facilitates their degradation or inhibits their translation [8]. After their initial identification in 1993 by Lee et al [9], the study of miRs has revealed new mechanisms for the regulation of gene expression and provided new directions in cancer research. MiRs are involved in a variety of biological functions, including cellular proliferation and cell cycle control, apoptosis, angiogenesis, tissue invasion, and metastasis, suggesting that they have a vital role in the development and progression of different cancers [10–12]. Correspondingly, miRs have also been shown to have prognostic significance in several tumor types, including colon [13], lung [14], breast [15], and ovarian cancer [16]. Recent studies have shown that miRs are also potential prognostic factors in RCC, suggesting that they could be developed as prognostic biomarkers to guide therapeutic decisions [17–19].

To date there has only been one published study evaluating the prognostic value of different miRs in RCC [7], and this did not follow the MOOSE or PRISMA guidelines, and had only a limited analysis of prognosis. Therefore, we conducted a systematic review of studies that have identified a relationship between miR expression and survival in RCC, and included these in a meta-analyses if the extracted data could be merged.

## RESULTS

### Selection of studies

**Figure 1:**
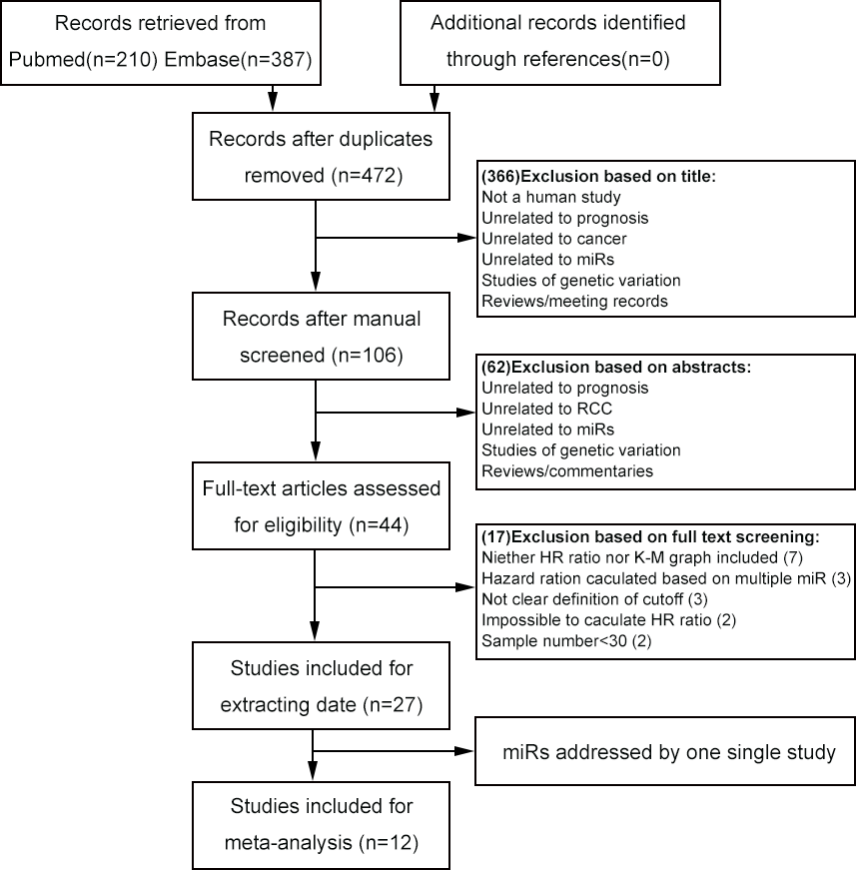
Flowchart of selecting studies for inclusion

A total of 597 records were retrieved from the primary literature of the below databases. A total of 125 duplicate reports were excluded. After screening the titles of 597 studies returned from the initial search strategy, the abstracts of 106 of these studies were reviewed. This left 44 articles that met the inclusion criteria. After screening the titles, abstracts, publication types, and full texts of these articles, 27 studies were included in the present study and used for data extraction (Figure 1 and Table 1). We then examined whether a sufficient number of these 27 studies pertained to specific miRs to allow a meta-analysis to be conducted. Finally, a total of 12 publications addressing the relationship between four specific miRs (miR-21, miR-126, miR-210, and miR-221) and RCC were found to meet all of the inclusion criteria. These also provided the total data set for the meta-analysis [20–31]. All of the selected studies were nonrandomized. A flowchart of the study selection process is shown in Figure 1.

**Table 1:** The main characteristics of enrolled studies

Study (year)	miR	Population	Study design	Stage	Case number	Assay method	Cut-off	Detected sample	Survival analysis	Source of HR	Adjusted	Follow up(month)
Neal 2010	210	Australia	R	pT1–pT4	31	qRT-PCR	Maximum NT value	Tissue	OS	DE/SC	–	∼140
Slaby 2010	106b	Czech	R	T1–T3	38	qRT-PCR	Median	Frozen tissue	RFS	SC	–	3∼105
Cui 2012	99a	China	R	T1–T4	40	qRT-PCR	T/N ratio < 0.5	Frozen tissue	OS	SC	–	∼70
Faragalla 2012	21	Canada	R	T1–T3	88	qRT-PCR	40th percentile	Tissue	OS, DFS	Rep	Yes	∼192
Hirata 2012	1826	Japan	R	T1–T4	46	qRT-PCR	Median	Tissue	OS, RFS	SC	–	∼120
Zaman 2012	21	USA	R	–	36	qRT-PCR	T/N ratio > 1.2	FFPE	OS	SC	–	∼60
Goto 2013	486	Japan	R	I–IV	150	qRT-PCR	Quartile	FFPE	CSS	SC	–	2–120
	486	Japan	R	III–IV	46	qRT-PCR	Quartile	FFPE	CSS	Rep	Yes	2–120
Hirata 2013	1260b	Japan	R	pT1–pT4	43	qRT-PCR	Median	FFPE	OS	SC	–	∼110
Khella 2013	215	Canada	R	–	218	qRT-PCR	X-tile algorithm	Tissue	OS	SC	–	∼100
Li 2013	217	China	R	T1–T4	44	qRT-PCR	T/N ratio < 0.49	Frozen tissue	OS	DE/SC	–	∼60
McCormick 2013	210	UK	R	T1–T3	46	qRT-PCR	Median	Tissue	OS	Rep	–	∼100
Shinmei 2013	155	Japan	R	I–IV	137	qRT-PCR	Median	FFPE	CSS	SC	–	2∼188
	155	Japan	R	III–IV	43	qRT-PCR	Median	FFPE	CSS	SC	–	2∼188
Wang 2013	100	China	R	T1–T4	96	qRT-PCR	Median	Frozen tissue	OS, CSS	Rep	Yes	25∼134
Wotschofsky 2013	210	Germany	R	pT1–pT4	87	qRT-PCR	Median	Frozen tissue	RFS	Rep	–	∼80
Zhao 2013	187	China	R	T1–T4	86	qRT-PCR	T/N ratio < 0.42	Frozen tissue	OS	DE/SC	–	∼60
Chen 2014	129–3p	China	R	pT1–pT4	69	qRT-PCR	Median	Tissue	OS, DFS	Rep	–	∼44
Fu 2014	125b	China	R	I–IV	276	ISH	X-tile program	FFPE	CSS, RFS	Rep	Yes	∼120
Ishihara 2014	23b	Japan	R	pT1–pT4	61	qRT-PCR	Median	Tissue	OS	SC	–	∼108
	27b	Japan	R	pT1–pT4	61	qRT-PCR	Median	Tissue	OS	SC	–	∼108
Teixeira 2014	221	Portugal	R	T1–T3	43	qRT-PCR	Quartile	Plasma	OS, CSS	SC/Rep	–/Yes	∼130
	222	Portugal	R	T1–T3	43	qRT-PCR	Quartile	Plasma	OS	SC	–	∼130
Vergho 2014	21	Germany	R	pT1–pT3	103	qRT-PCR	ROC curve	Frozen tissue	CSS	Rep	Yes	∼68
	126	Germany	R	pT1–pT3	103	qRT-PCR	ROC curve	Frozen tissue	CSS	SC	–	∼68
Vergho 2014	21	Germany	R	T3	37	qRT-PCR	ROC curve	FFPE	CSS	Rep	–	∼152
	126	Germany	R	T3	37	qRT-PCR	ROC curve	FFPE	CSS	Rep	–	∼152
	210	Germany	R	T3	37	qRT-PCR	ROC curve	FFPE	CSS	Rep	–	∼152
	221	Germany	R	T3	37	qRT-PCR	ROC curve	FFPE	CSS	Rep	–	∼152
Zhao 2014	630	China	R	T1–T4	92	qRT-PCR	Mean	Frozen tissue	OS	Rep	Yes	–
Ge 2015	210	USA	R	I–IV	58	Microarray	Median	Tissue	OS, RFS	SC/Rep	–/Yes	31.5∼86.1
Khella 2015	126	Canada	R	I–IV	260	qRT-PCR	X-tile algorithm	Tissue	OS, DFS	Rep	Yes	∼120
	126	USA	R	T1b-	268	qRT-PCR	X-tile algorithm	Tissue	OS	Rep	–	∼120
Samaan 2015	210	Canada	R	I–IV	262	qRT-PCR	X-tile algorithm	Tissue	OS, DFS	Rep	Yes	∼120
Tang 2015	21	China	R	–	45	qRT-PCR	X-tile algorithm	Frozen tissue	CSS	Rep	Yes	∼58.4
	210	China	R	–	45	qRT-PCR	X-tile algorithm	Frozen tissue	CSS	Rep	Yes	∼58.4
Zhao 2015	497	China	R	T1–T4	86	qRT-PCR	Mean	Frozen tissue	OS	Rep	Yes	∼60

HR = hazard ratio; MiR = microRNA; R = Retrospective; qRT-PCR = quantities reverse transcription polymerase chain reaction; ISH = *in situ* hybridization; FFPE = formalin-fixed, paraffin-embedded; OS = overall survival; RFS = recurrence free survival; DFS = disease free survival; CSS = cancer specific survival; DE = data extrapolated; SC = survival curve; Rep = Reported; –, not reported.

### Characteristics of the included studies

All of the included studies were published recently (2010–2015). They had a retrospective design, and reported the prognostic value of 21 different miRs in RCC patients, with a median sample size of 86 patients (range, 31–276 patients). Quantitative real-time PCR was used by most of the studies to measure miR expression, whereas *in situ* hybridization and microarray was used by only one study. MiR expression was mainly detected in tissue samples, while one study tested for miRs in plasma. In 12 studies, the hazard ratio (HR) was adjusted for other associated variables (covariates) including tumor site and size, patient age, tumor grade, and patient stage (Table 1).

### MiRs and prognosis

**Figure 2:**
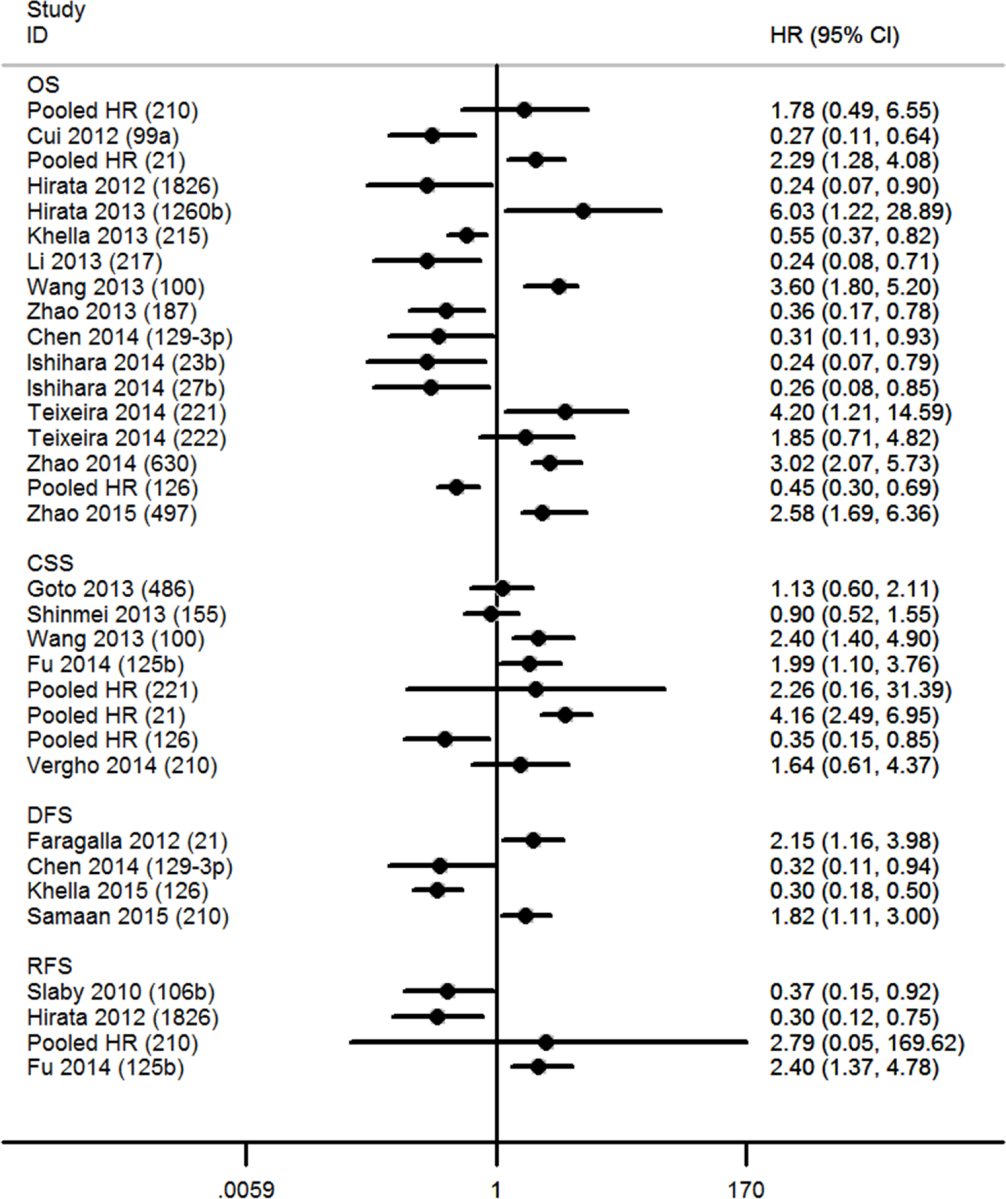
Hazard ratios (HR) of miRs The point estimate is bounded by a 95% confidence interval (CI), and the perpendicular line represents no increased risk for the outcome. OS = overall survival; CSS = cancer specific survival; DFS = disease free survival; RFS = recurrence free survival.

Increased expression of miR-21 [21, 22, 26, 27, 31], miR-1260b [32], miR-210 [28, 30, 31], miR-100 [33], miR-125b [34], miR-221 [25, 27], miR-630 [35], and miR-497 [36] were associated with a poor prognosis, as was the decreased expression of miR-106b [37], miR-99a [38], miR-1826 [39], miR-215 [40], miR-217 [41], miR-187 [42], miR-129–3p [43], miR-23b [44], miR-27b [44], and miR-126 [26, 27, 29] (Table 2 and Figure 2). MiR-222 expression did not show any significant association with cancer survival [25]. Although the expression of miR-486 [45] and miR-155 [46] was associated with the survival outcome of patients with stage III or IV RCC, it was not significantly associated with the prognosis of patients with all-stage RCC.

**Table 2:** Hazard ratios for microRNAs

Study	miR	Case number		OS		CSS/DFS		RFS		Expression associates with bad prognosis
		High level	Low level	HR (95% CI)	*P*	HR (95% CI)	*P*	HR (95% CI)	*P*	
Neal 2010	210	17	14	2.41(0.65–8.96)	0.189	–	–	–	–	High
Slaby 2010	106b	19	19	–	–	–	–	0.37(0.15–0.92)	0.032	Low
Cui 2012	99a	11	29	0.27(0.11–0.64)	0.003	–	–	–	–	Low
Faragalla 2012	21	48	40	1.97(1.04–3.73)	0.036	2.15 (1.16–3.98)^D^	0.014	–	–	High
Hirata 2012	1826	23	23	0.24(0.07–0.90)	0.0347	–	–	0.30(0.12–0.75)	0.0104	Low
Zaman 2012	21	30	6	4.50(1.16–17.49)	0.030	–	–	–	–	High
Goto 2013	486	112	38	–	–	1.13(0.60–2.11)^C^	0.7062	–	–	High
	486	34	12	–	–	4.33(1.45–18.71)^C^	0.0064	–	–	High
Hirata 2013	1260b	21	22	6.03(1.22–28.89)	0.0278	–	–	–	–	High
Khella 2013	215	165	53	0.55(0.37–0.82)	0.0032	–	–	–	–	Low
Li 2013	217	9	34	0.24(0.08–0.71)	< 0.01	–	–	–	–	Low
McCormick 2013	210	23	23	0.33(0.15–0.72)	0.005	–	–	–	–	Low
Shinmei 2013	155	69	68	–	–	0.90(0.52–1.55)^C^	0.7001	–	–	Low
	155	21	22	–	–	0.47(0.23–0.94)^C^	0.0337	–	–	Low
Wang 2013	100	60	36	3.6(1.8–5.2)	0.01	2.4(1.4–4.9)^C^	0.02	–	–	High
Wotschofsky 2013	210	43	44	–	–	–	–	0.39(0.12–1.23)	0.109	Low
Zhao 2013	187	18	68	0.36(0.17–0.78)	< 0.01	–	–	–	–	Low
Chen 2014	129–3p	–	–	0.31(0.11–0.93)	0.037	0.32(0.11–0.94)^D^	0.039	–	–	Low
Fu 2014	125b	–	–	–	–	1.99(1.10–3.76)^C^	0.024	2.40(1.37–4.78)	0.005	High
Ishihara 2014	23b	31	30	0.24(0.07–0.79)	0.0183	–	–	–	–	Low
	27b	31	30	0.26(0.08–0.85)	0.0253	–	–	–	–	Low
Teixeira 2014	221	11	32	4.20(1.21–14.59)	0.024	10.7(1.33–85.65)^C^	0.026	–	–	High
	222	11	32	1.85(0.71–4.82)	0.208	–	–	–	–	High
Vergho 2014	21	43	60	–	–	6.47(1.84–22.73)^C^	0.0008	–	–	High
	126	31	72	–	–	0.20(0.07–0.58)^C^	0.0032	–	–	Low
Vergho 2014	21	–	–	–	–	3.52(1.93–6.44)^C^	0.0001	–	–	High
	126	–	–	–	–	0.50(0.28–0.87)^C^	0.012	–	–	Low
	210	–	–	–	–	1.14(0.91–1.44)^C^	0.231	–	–	High
	221	–	–	–	–	0.71(0.45–1.14)^C^	0.139	–	–	Low
Zhao 2014	630	58	34	3.02(2.07–5.73)	0.016	–	–	–	–	High
Ge 2015	210	29	29	6.50(1.76–24.00)	0.005	–	–	26.01(2.42–279.1)	0.007	High
Khella 2015	126	210	50	0.40(0.19–0.86)	0.019	0.30(0.18–0.50)^D^	< 0.001	–	–	Low
	126	–	–	0.48(0.29–0.80)	0.0035	–	–	–	–	Low
Samaan 2015	210	112	150	2.46(1.20–5.04)	0.014	1.82 (1.11–3.00)^D^	0.018	–	–	High
Tang 2015	21	–	–	–	–	6.46(1.35–30.94)^C^	0.02	–	–	High
	210	–	–	–	–	3.27(1.01–10.59)^C^	0.05	–	–	High
Zhao 2015	497	38	48	2.58(1.69–6.36)	< 0.001	–	–	–	–	High

OS = overall survival; CSS = cancer specific survival; DFS = disease free survival; RFS = recurrence free survival; HR = hazard ratio;

C CSS

D DFS.

**Figure 3:**
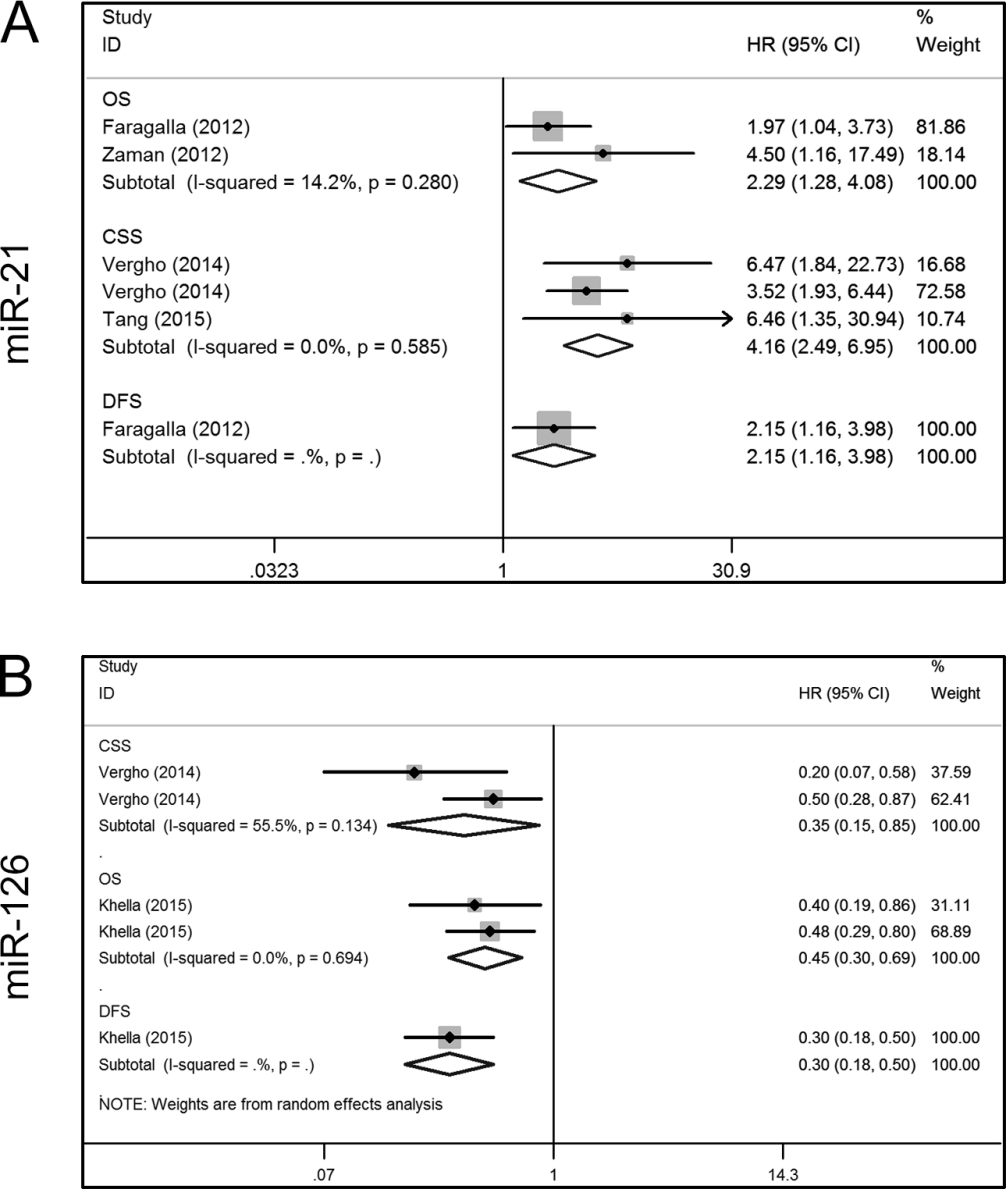
Forest plots of studies evaluating hazard ratios of aberrant miR-21 and mir-126 expression **A.** miR-21, OS, CSS, DFS; **B.** miR-126, CSS, OS, DFS. OS = overall survival; CSS = cancer specific survival; DFS = disease free survival; HR = hazard ratio.

Four miRs (miR-21, miR-126, miR-210 and miR-221) were investigated by at least two studies, and we conducted meta-analyses of the corresponding data. Five articles included survival data for miR-21, two articles contained OS data [21, 22], three had CSS data [26, 27, 31], and one had DFS data [21]. When we performed a meta-analysis on the relationship of miR-21 expression and the OS and CSS of RCC patients, no significant heterogeneity was found (OS, *I*^2^ = 14.2%, *P* = 0.280; CSS, *I*^2^ = 0.0%, *P* = 0.585), and the fixed-effect model was therefore applied. This revealed that a higher miR-21 expression was predictive of shorter OS (HR, 2.29; 95% confidence interval [CI], 1.28–4.08; *P* = 0.005) and CSS (HR, 4.16; 95% CI, 2.49–6.95; *P* < 0.001). Faragalla et al. [21] also reported a shorter DFS in RCC patients with an elevated level of miR-21 (HR, 2.15; 95% CI, 1.16–3.98; *P* = 0.014) (Figure 3A). Three articles addressed the role of miR-126 in the survival outcome of RCC patients, two of which focused on CSS [26, 27], and one focused on OS and DFS [29]. There was significant heterogeneity among the selected studies with respect to CSS (*I*^2^ = 55.5%, *P* = 0.134), and thus a random-effect model was used. The results showed that lower miR-126 expression predicted a shorter CSS (HR, 0.35; 95% CI, 0.15–0.85; *P* = 0.019). Unlike CSS, there was no significant heterogeneity in OS (*I*^2^ = 0.0%, *P* = 0.694), and hence a fixed-effect model was applied. This indicated that lower miR-126 expression was associated with shorter OS in RCC (HR, 0.45; 95% CI, 0.30–0.69; *P* < 0.001). In addition, Khella et al. [29] reported shorter DFS (HR, 0.30; 95% CI, 0.18–0.50; *P* < 0.001) in RCC patients with reduced miR-126 expression (Figure 3B).

**Figure 4:**
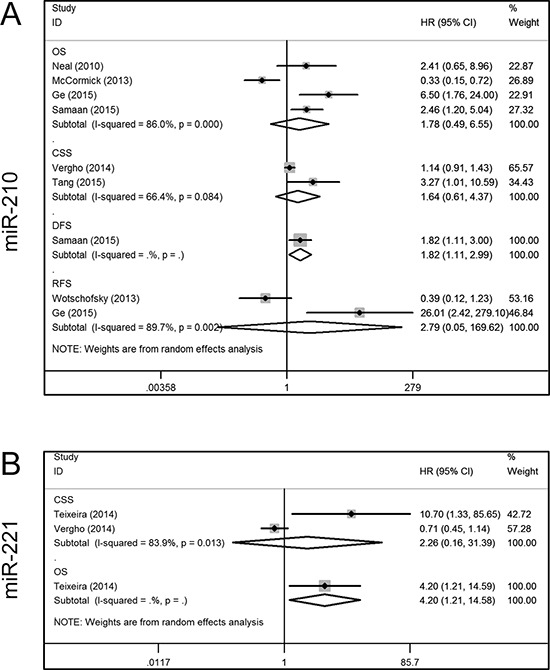
Forest plots of studies evaluating hazard ratios of aberrant miR-210 and mir-221 expression **A.** miR-210, OS, CSS, DFS, RFS; **B.** miR-221, CSS, OS. OS = overall survival; CSS = cancer specific survival; DFS = disease free survival; RFS = recurrence free survival; HR = hazard ratio.

Seven articles addressed the relationship between miR-210 expression and the prognosis of RCC patients, of which four included OS data [20, 23, 28, 30], one included data on DFS [30], and two included data on CSS [27, 31] and RFS [24, 28]. Because of significant inter-study heterogeneity, a random-effect model was applied in the analysis (*I*^2^ = 86.0%, *P* = 0.000). This revealed that increased miR-210 expression tended to occur in patients with a shorter OS, although this was not statistically significant (HR, 1.78; 95% CI, 0.49–6.55; *P* = 0.162). Additionally, Samaan et al. [30] found that there was a shorter DFS in patients with elevated miR-210 levels (HR, 1.82; 95% CI, 1.11–2.99; *P* = 0.018). However, miR-210 expression was not found to be related to CSS (HR, 1.64; 95% CI, 0.61–4.37; *P* = 0.138) or RFS (HR, 2.79; 95% CI, 0.05–169.62; *P* = 0.810) (Figure 4A).

For miR-221, one study included OS and CSS data [25], and one contained only CSS data [27]. A random-effect model was used due to significant inter-study heterogeneity (*I*^2^ = 83.9%, *P* = 0.013), and revealed that aberrant miR-221 expression was not related to CSS (HR, 2.26; 95% CI, 0.16–31.39; *P* = 0.543). In addition, Teixeira et al. [25] reported that an increased plasma level of miR-221 was associated with shorter OS (HR, 4.20; 95% CI, 1.21–14.58; *P* = 0.024) (Figure 4B).

Due to the small size of this study, no conclusive graph could be generated, and we therefore did not evaluate publication bias.

## DISCUSSION

Over the past decade, there has been increasing evidence that aberrant expression of several miRs is associated with survival outcome in cancer patients [47–50]. MiRs are also known to play key roles in the pathogenesis of cancer, as the up-regulation of oncogenic miRs or the down-regulation of cancer suppressive miRs can contribute to tumorigenesis through effects on many cellular process, including the cell cycle, angiogenesis, invasion, and metastasis [51, 52].

In response to the need for independent prognostic molecular markers for RCC that are readily assayable on routinely acquired clinical specimens, we conducted this comprehensive systematic review and meta-analysis of the current literature on RCC, evaluated the inconsistencies between these reports, and undertook a comprehensive assessment of the prognostic value of miRs. Our study is the first extensive report focusing on this association, in which 27 studies involving 2578 subjects were analyzed and 21 miRs involved in the survival analysis of RCC were compared.

In the current study, we found that the elevated expression of miR-21 [21, 22, 26, 27, 31], miR-1260b [32], miR-210 [28, 30, 31], miR-100 [33], miR-125b [34], miR-221 [25, 27], miR-630 [35], and miR-497 [36] were associated with poor survival in RCC patients, whilst the decreased expression of miR-106b [37], miR-99a [38], miR-1826 [39], miR-215 [40], miR-217 [41], miR-187 [42], miR-129–3p [43], miR-23b [44], miR-27b [44], and miR-126 [26, 27, 29] were likewise associated with a worse prognosis.

Although a number of miRs were found to be associated with the prognosis of RCC patients, most of them were identified only by a single study, and only four miRs (miR-21, miR-126, miR-210 and miR-221) were reported by at least two studies. We therefore performed the meta-analyses on these four miRs and merged the data. The results indicate that an elevated miR-21 level predicts poor survival in RCC patients, who are likely to have shorter OS, CSS, and DFS. Conversely, a lower expression level of miR-126 predicts worse CSS, OS, and DFS in RCC patients. Furthermore, increased expression of miR-210 is associated with shorter DFS, and an elevated plasma level of miR-221 is associated with shorter OS.

MiR-21 is one of the most extensively studied cancer-related miRs and might be the most relevant oncogenic factor in most cancers [53–55]. Increased miR-21 expression enhances tumor growth, migration, and invasion, and reduces sensitivity to chemotherapy through modulating various target genes [21, 22, 56]. Cancer patients with higher miR-21 expression levels always suffered from a poorer prognostic outcome [57], which is consistent with our findings. There are a number of molecular mechanisms that could explain this relationship. Dey et al. [58] showed that miR-21 mediated the post-transcriptional regulation of phosphatase and tensin homolog (PTEN) that in turn increased canonical oncogenic Akt/TORC1 signaling to drive renal cancer cell proliferation and invasion. Furthermore, miR-21 promoted renal cancer cell hyperplasia and contributed to tumor cell transformation and metastasis, but also post-transcriptionally down-regulated the expression of the PDCD4 tumor suppressor gene [59]. MiR-126 is located in intron 7 of the epidermal growth factor-like protein 7 gene (EGFL7) on chromosome 9 [29], and is down regulated in various cancer types including breast, gastric, and prostate cancer, and RCC [26]. In the latter, miR-126 features in the molecular classification of different tumor sub-types [60]. More recently, miR-126 down-regulation has been linked to RCC progression, and has been shown to act as a tumor suppressor in various cancer types including RCC through regulating target genes such as CRK, VEGF, and EGFL7 in cancer cells [61, 62]. Khella et al. [29] found that miR-126 is down-regulated in metastatic compared to primary ccRCC, and in tumors with a higher stage or grade. Their target prediction and pathway analysis showed that miR-126 can regulate key molecules and critical pathways involved in ccRCC tumor development and progression, including the IGF1R, BCL2, HIF-1, VEGF, mTOR, and PI3K-Akt signaling pathways.

Hypoxia is an important pathophysiological process in solid cancers including RCC, and has been shown to influence miR-210 expression. MiR-210 is up-regulated in renal cancer [63–65], and is included in a miR-based classification system of this disease [66]. In ccRCC, VHL gene mutations lead to the up-regulation of HIF-1 and HIF-2, with subsequent overexpression of miR-210 [23]. Conversely, miR-210 was also shown to regulate HIF-1 protein and other target genes in RCC, affecting carcinogenesis-related processes such as cell migration and invasion, cell survival, apoptosis, mitochondrial metabolism, angiogenesis, and DNA repair [67, 68]. In addition, studies have shown that miR-210 targets the iron sulfur cluster homologue gene (ISCU), the product of which acts as a scaffold protein for the formation of iron sulfur (Fe–S) clusters [20, 23]. However, reports of an association between increased miR-210 expression and prognosis in RCC are inconsistent [20, 23, 24, 27, 28, 30, 31], and hence larger, multicenter studies are needed to address this.

Recent studies have reported tumor-specific miR-221 expression that is mediated by intricate regulatory mechanisms, and several target genes of miR-221 influence tumorigenesis and progression. These include the cell cycle regulators p27Kip1 and p57Kip2, which are repressed by miR-221 in multiple cancers to induce tumor cell proliferation. MiR-221 also directly inhibits the post-transcriptional expression of metallopeptidase inhibitor 3 (TIMP3), an inhibitor of matrix metalloproteinases (MMPs), and plays an important role in promoting the invasion of human gliomas. The oncogenic effect of miR-221 is also mediated by PTEN [69]. In renal tumors, mir-221 is included in a miR-based classification system [66]. Its expression is activated by EGFR signaling, and it can subsequently modulate metastasis in RCC. Teixeira et al. [25] found that plasma miR-221 levels varied during RCC development, and that increased levels were associated with a shorter OS.

In addition to the above, we also systematically investigated the relationship of other miRs with RCC prognosis in this study. These relationships, as well as the possible roles of other miRs in RCC progression are summarized in Figure 2 and Table 3.

**Table 3:** Summary of miRs with altered expression, their potential targets and pathways entered this study

microRNA	Expression	Potential target	Pathway	Reference
miR-210	Up	ISCU1/2, HIF	VHL/HIF/hypoxia pathway	20,23,24,27,28,30,31
miR-21	Up	P53, TGF-β, VHL, EGLN1, PTEN, TSC1, TSC2, PDCD4,	Cell cycle control/proliferation, migration and invasion, mitochondrial apoptosis pathway	21,22,26,27,31
miR-486	Up	PTEN, OLFM4, FOXO1	Cell adhesion and migration	45
miR-1260b	Up	sFRP1, Dkk2, Smad4	Cell proliferation, apoptosis, invasion	32
miR-100	Up	mTOR	Cell proliferation	33
miR-125b	Up	E2F3, P53, BAK1, MMP9	Cell growth, apoptosis, metastasis	34
miR-221	Up	PTEN, TIMP3, c-Kit, p21, p53, p57	Cell cycle, proliferation, apoptosis, migration, invasion, EMT, EGFR signaling pathway	25,27
miR-222	Up	PTEN, TIMP3, c-Kit, p21	Cell cycle, migration, invasion, EMT	25
miR-630	Up	IGF-1R	Cell death and apoptosis	35
miR-497	Up	IGF-1R	Cell proliferation, migration, invasion	36
miR-106b	Down	TGF-β signaling	TGF-β signaling	37
miR-99a	Down	mTOR	Cell growth/cycle control, migration and invasion, mTOR pathway	38
miR-1826	Down	CTNNB1, MEK1	Cell proliferation, invasion, migration, apoptosis	39
miR-215	Down	MDM2, ZEB2, TYMS	Cell migration, invasion, proliferation	40
miR-217	Down	SirT1, KRAS	Cell proliferation, migration	41
miR-155	Down	SOCS1, SHIP1, TP53INP1	Apoptosis-related signaling, hypoxia pathway	46
miR-187	Down	B7-H3, Dab2	Cell growth, migration, EMT	42
miR-129–3p	Down	SOX4, p-FaK, MMP2, MMP9	Cell migration, invasion	43
miR-23b/27b	Down	cytokine interaction pathway	Cell proliferation, migration and invasion	44
miR-126	Down	SPRED1, IGF1R, BCL2, CRK, CCNE2, PIK3R2, EGFL7	Apoptosis, HIF-1, VEGF, mTOR, and PI3K-Akt signaling pathways	26,27,29

HIF = hypoxia inducible factor; PTEN = phosphatase and tensin homologue; TGF = transforming growth factor; mTOR = mammalian target of rapamycin; TSC = tuberous sclerosis; MMP = matrix metalloproteinase; TIMP = tissue inhibitor of metalloproteinase; EMT = epithelial-to-mesenchymal transition; EGFR = epidermal growth factor receptor; IGF = insulin-like growth factor; VEGF = vascular endothelial growth factor.

Some limitations need be considered in the interpretation of the results of the current study. First, although 27 studies involving 2578 patients were included in this systematic review, most of them addressed diverse miRs and used different follow-up endpoints. Only four miRs (miR-21, miR-126, miR-210, and miR-221) were identified by at least two studies, and these also used different outcome assessments. Thus, most of meta-analyses in our study only contain two or three records, and large prospective studies are therefore needed to confirm our findings and allow rigorous conclusions to be made. Further, due to inadequate data, we did not evaluate publication bias. The lack of these analyses may partly affect the interpretation of the results and make them less reliable. Second, marked study heterogeneity was seen in some analyses. The heterogeneity of the subjects was probably due to differences in factors such as the patients’ baseline characteristics (ethnicity, nationality, gender, age, and tumor stage and grade), different assay methods, diverse cut-off values for miR expression, the way samples were prepared and preserved (i.e. paraffin fixed, formalin fixed, freshly frozen tumors or blood), treatment, and duration of follow-up. The method of extracting the HRs of these studies may also have introduced heterogeneity. Seven of the studies included in the systematic review did not report an HR or other survival related statistics. Therefore, we had to extract the required data using a Kaplan-Meier graph that was prone to error, even when two independent reviewers extracted the data. The calculated HRs may thus not be as dependable as those retrieved directly from reported statistics. Finally, a number of unavoidable limitations exist. All meta-analyses are affected by the quality of their component studies; the fact that research with statistically significant results is potentially more likely to be submitted and published than work with null or non-significant results could compromise the validity of such analyses [70]. Furthermore, the current meta-analysis of published studies does not have the benefit of currently unpublished data [71]. Recently, circulating markers have become more acceptable than tissue markers because they can be evaluated before surgery and be monitored throughout the life of the patient. Further studies are needed to establish the prognostic value of miR serum levels [25]. It should be noted that some studies developed combined expression signatures of multiple miRs [19, 72, 73], which requires a robust validation strategy. Furthermore, the model has to be validated in a separate experiment using an independent patient cohort. Hence, developing a new molecular signature by using diverse miRs and investigating their efficacy may be useful.

Despite the limitations described above, our comprehensive systematic review and meta-analysis reveals that miRs, especially miR-21 and miR-126, could be promising, convenient and potentially non-invasive prognostic markers in RCC, and could allow monitoring for cancer progression or recurrence. These miRs may also be useful therapeutic targets in RCC. However, more studies are needed to clarify the prognostic value of these novel biomarkers and address possible discrepancies.

## MATERIALS AND METHODS

This meta-analysis was conducted following the guidelines of the Meta-analysis of Observational Studies in Epidemiology group (MOOSE) [74] and Preferred Reporting Items for Systematic Reviews and Meta-analysis (PRISMA) criteria [75].

### Search strategy

A literature search was performed on Pubmed and Embase for studies that analyzed associations between miRs and prognosis in RCC patients on April 15, 2015, using the following search strategy: (microRNA OR miRNA OR miR) AND (cancer OR tumor OR neoplasm OR malignant OR metastasis OR carcinoma OR renal cell carcinoma OR RCC) AND (renal or kidney) AND (prognosis OR prognostic OR survival OR outcome OR mortality). Additionally, we manually screened the references from the relevant literature, including all of the identified studies, reviews, and editorials.

### Eligibility criteria

The main criteria considered in including a study were investigating the prognosis of RCC, measuring the expression of specific miRs in tissue or serum and studying their association with survival outcome. Survival outcome was further explored considering Hazard ratio (HR) with Confidence interval (CI), HR with *P* value, Kaplan–Meier curves or obtaining the required data by contacting correspondent author. Articles were excluded if they (1) were not written in English; (2) were case reports, letters, commentaries, meeting records or review articles; (3) had sample of fewer than 30 cases; (4) concerned genetic alterations of a miR including polymorphisms or methylation patterns; (5) calculated hazard ratios (HRs) based on multiple miR; (6) lacked sufficient data for estimating HRs and their 95% confidence intervals (CIs). When duplicate studies were retrieved, we included the most informative and recent article.

Thereafter, the papers that fulfilled all selection criteria were processed for data extraction. Three individual researchers (L. Gu., H. Li., and L. Chen) independently assessed eligibility of the retrieved articles. Discrepancies were resolved by discussion.

### Quality assessment

The quality of all the included studies was systematically assessed according to the following checklist based on the Dutch Cochrane Centre represented by MOOSE for epidemiologic studies [74]: (1) clearly defined study design; (2) clearly described study population (Country); (3) sufficiently large sample (*N* > 30 for the current study); (4) clearly described outcome assessment by representing it in overall survival (OS), cancer-specific survival (CSS), disease-free survival (DFS) or recurrence-free survival (RFS); (5) clear definition of measurement of miR (quantitative real-time polymerase chain reaction (qRT-PCR) (TaqMan assay or stem-loop primer assay) or *in situ* hybridization (ISH), hybridized oligonucleotide microarray (oligoarray)); (6) clear definition of cut-off; and (7) sufficiently long follow-up.

To assure the quality of this meta-analysis, studies were excluded if they do not meet these seven criteria.

### Data extraction

Data were extracted independently by three investigators (L. Gu., H. Li., and L. Chen), who used a predefined sheet to retrieve information about all studies that qualified for final inclusion. Data sheets were designed according to previous studies focusing on similar issue and PRISMA guideline [49, 75]. The following data were extracted: (1) publication information: first author's last name, year of publication and study design; (2) patients’ characteristics: population study, investigated microRNAs, number of participants, stage of disease, detected sample and follow-up duration; (3) miR expression measurement and cut-off value; and (4) HRs of elevated miRs for OS, CSS, DFS, RFS, as well as their 95% CIs and *P* values. If available, the HRs with their 95% CIs and *P* values were collected from the original article or the correspondent author. If not, we calculated HRs and their 95% CIs using the data of observed events, the data of samples in each group or the data provided by the authors. If only Kaplan–Meier curves were available, we extracted data from the graphical survival plots and estimated the HRs. All the calculations mentioned above were based on the methods illustrated by Parmar et al. [76] and Tierney et al [77].

### Statistical analysis

A test of heterogeneity of combined HRs was conducted using Cochran's Q test and Higgins *I*-squared statistic. A *P* value of less than 0.1 was considered significant. *I*^2^ values of >50% indicate heterogeneity among studies. A random effect model was applied if heterogeneity was observed (*P* < 0.1), while the fixed effect model was used in the absence of between-study heterogeneity (*P* > 0.1). An observed HR > 1 implied a worse survival for the group with elevated miR expression. Conversely, an observed HR < 1 implied a worse survival for the group with decreased miR expression. We pooled HRs of the studies by using Stata 12.0 software (StatCorp, College Station, TX, USA).
